# Comparison of guidelines for the management of rectal cancer

**DOI:** 10.1002/bjs5.88

**Published:** 2018-07-27

**Authors:** E. Luzietti, G. Pellino, S. Nikolaou, S. Qiu, S. Mills, O. Warren, P. Tekkis, C. Kontovounisios

**Affiliations:** ^1^ Department of Surgical Sciences Azienda Ospedaliero – Universitaria di Parma Parma Italy; ^2^ Department of Medical, Surgical, Neurological, Metabolic and Ageing Sciences Universtià della Campania ‘Luigi Vanvitelli’ Naples Italy; ^3^ Department of Colorectal Surgery Royal Marsden Hospital London UK; ^4^ Department of Surgery and Cancer Imperial College London London UK; ^5^ Department of Colorectal Surgery Chelsea and Westminster Hospital London UK

## Abstract

A comparison between NCCN, ESMO and JSCCR Guidelines is presented, concerning the treatment of rectal cancer, with an analysis and discussion of their discrepancies.

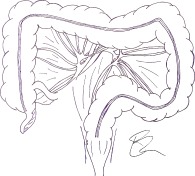

Differences indicate areas for research

## Abstract (it)

**Background:**

Overall survival from rectal cancer has almost doubled over the last 20 years. Following recommendations in management guidelines plays some part in this, but the extent of discrepancies between them has not been evaluated.

**Methods:**

National Comprehensive Cancer Network (NCCN, USA), European Society for Medical Oncology (ESMO, Europe) and Japanese Society for Cancer of the Colon and Rectum (JSCCR, Japan) guidelines were examined and compared. These were chosen as representative of the countries with the highest incidences of rectal cancer and because no previous collaborations among societies were found.

**Results:**

Overall agreement among societies was found regarding the definition of total mesorectal excision as the surgical standard, the administration of adjuvant therapy for stage III disease, indications for surgical resection for metastases and/or recurrent disease, and the treatment of peritoneal disease. Discrepancies emerged, in particular between Western and Japanese guidelines. The most significant differences involved the endoscopic approach to early cancer, extended lymph node dissection, adjuvant treatment for patients with stage I and II disease, neoadjuvant chemotherapy, the specific management of metachronous disease, and restaging strategies.

**Conclusion:**

There are major discrepancies among guidelines. These differences should constitute topics for further research.

## Introduction

The 5‐year survival rate for rectal cancer approaches 60 per cent^1^. Although around a quarter of all patients present with metastases at initial diagnosis and half of all patients develop metastases^2^, the median overall survival for patients with metastatic disease is around 30 months, more than double that of 20 years ago. Factors that may have contributed to this improvement include closer follow‐up and earlier detection of recurrent disease, efficacy of systemic therapies, resection of metastases and implementation of a ‘continuum of care’^3^.

To standardize care, several organizations have created guidelines and protocols regarding the management of rectal cancer. Despite this, there remains a certain degree of variation among guidelines.

The aim of this study was to compare the guidelines for the management of rectal cancer from the European Society of Medical Oncology (ESMO, Europe)^1^, ^2^, ^3^, the National Comprehensive Cancer Network (NCCN, USA)^4^ and the Japanese Society for Cancer of the Cancer of Colon and Rectum (JSCCR, Japan)^5^, highlighting and analysing both agreements and discrepancies, and considering further developments. These guidelines were chosen because of the high incidence of rectal cancer in those countries and the lack of previous collaborative guidelines among those societies.

## Methods

NCCN, ESMO and JSCCR guidelines were included, with the relative position papers: NCCN Guidelines for Rectal Cancer version 3.2017^4^; ESMO Clinical Practice Guidelines for Diagnosis, Treatment and Follow‐up of rectal cancer (2013 edition)^1^ and metastatic colorectal cancer (2014 edition)^2^; ESMO consensus guidelines for the management of patients with metastatic colorectal cancer (2016 edition)^3^; and JSCCR guidelines 2016 for the treatment of colorectal cancer^5^. Guidelines were accessed at organizational websites or collected from available publications. Access was sought for guidelines that were not open‐access.

Outcome measures were agreements and discrepancies among NCCN, ESMO and JSCCR for each specific topic of rectal cancer management, and results are presented in sections.

## Results

Analyses included four journal articles^1^, ^2^, ^3^ ^5^ and one guideline document^4^. Both NCCN and ESMO guidelines discussed rectal cancer as a specific entity, with the exception of metastatic disease, which was considered in conjunction with colonic cancer. JSCCR guidelines presented colonic and rectal cancer without distinction.

There was some concordance between Western and Asian guidelines for some topics, such as the definition of total mesorectal excision (TME) as surgical standard, administration of adjuvant therapy for stage III disease, surgical resection for metastases and/or recurrent disease, and cytoreductive surgery followed by intraperitoneal chemotherapy for peritoneal carcinosis.

### Pedunculated or sessile polyp (adenoma) with invasive cancer

Guidelines from all three societies indicated the need for pathology review.

NCCN advises simple observation after adequate removal of pedunculated polyps; this approach is also considered for sessile polyps removed completely. Transabdominal resection or transanal excision (standing on specific criteria) is recommended in the case of fragmented specimens, unassessable margins and/or unfavourable histological features.

According to ESMO, the choice between local procedure or radical standard surgery with or without adjuvant treatment is based on Haggitt's level or Kikuchi's system^6^ ^7^, grading and vascular invasion.

JSCCR and ESMO recommend endoscopic management of pedunculated and sessile polyps (endoscopic mucosal resection (EMR) or endoscopic submucosal dissection (ESD) according to size), preferably located distally to the peritoneal reflection; or TME, considering depth of invasion, histology and budding (*Table* 1).

**Table 1:** Precancerous lesions and invasive cancer: diagnosis and surgery

Topic	NCCN	ESMO	JSCCR
Pedunculated or sessile polyp (adenoma) with invasive cancer*			
Work‐up	Pathology review Colonoscopy Marking of cancerous polyp at time of colonoscopy or within 2 weeks	Biopsy Palpation Rigid sigmoidoscopy (flexible endoscopy) Haggitt's subclassification (if stalked adenoma) Kikuchi (sm) system (if sessile adenoma) ERUS, MRI	Information on size, predicted depth of invasion and morphology of the tumour
Findings and primary treatment			
Pedunculated polyp with invasive cancer, completely removed, with favourable histological features and clear margins (T1 only)*	Observe	Haggitt 1–3, T1 sm1 (–2?) N0: Local procedure, e.g. transanal endoscopic microsurgery (TEM)	Intramucosal carcinoma (cTis) or carcinoma with slight submucosal invasion (cT1): Endoscopic polypectomy – up to 2 cm in size
Sessile polyp with invasive cancer, completely removed, with favourable histological features and clear margins (T1 only)*	Observe Transanal excision (if appropriate) Transabdominal resection		Intramucosal carcinoma (cTis) or carcinoma with slight submucosal invasion (cT1): Endoscopic mucosal resection (EMR) or using a cap (EMRC) – up to 2 cm size Endoscopic submucosal dissection (ESD)
Fragmented specimen or margin cannot be assessed or unfavourable histological features*	Transanal excision (if appropriate) Transabdominalresection	Haggitt 4, T1 sm ≥ 2, high‐grade, VI: Radical standard surgery: TME Chemoradiotherapy (if surgery contraindicated) Local radiotherapy as an alternative to local surgery, alone or with (preoperative) chemoradiotherapy	Depth of SM invasion ≥ 1000 μm Vascular invasion positive Poorly differentiated adenocarcinoma, signet‐ring cell carcinoma or mucinous carcinoma Grade 2/3 budding at the site of deepest invasion Surgical resection: TME
Additional comments	Criteria for transanal excision†: < 30% circumference of bowel < 3 cm in size Margin clear (> 3 mm) Mobile, non‐fixed Within 8 cm of anal verge T1 only Endoscopically removed polyp with cancer or indeterminate pathology No lymphovascular or perineural invasion Well to moderately differentiated No evidence of lymphadenopathy on pretreatment imaging	Haggitt's levels 1–3 correspond to sm1 Haggitt's level 4 may be sm1–3	Local excision is indicated for cancers located distal to the second Houston valve (peritoneal reflection)
Rectal cancer (appropriate for resection)‡			
Work‐up	Biopsy Pathology review Colonoscopy CBC, chemistry profile, CEA Chest–abdomen–pelvis CT Pelvic MRI with contrast (preferred) or endorectal US Enterostomal therapist counselling PET‐CT only if equivocal CT findings or contraindications to intravenous contrast	Biopsy Palpation Rigid sigmoidoscopy (flexible endoscopy) ERUS, MRI CRM evaluation CT, MRI (or US) of liver/abdomen CT/chest X‐ray of thorax MDT conference	Not formally stated
Findings and management			
Resectable‡	T1–2 N0: transanal excision (if appropriate) or transabdominal resection‡ Tany Nany M1 resectable metastases: neoadjuvant treatment (combination chemotherapy or infusional 5‐FU/pelvic RT or capecitabine/RT or bolus 5‐FU + leucovorin/pelvic RT or short‐course RT (not recommended for T4)) followed by primary^**^ and adjuvant treatment§	cT1–2; cT3a (b) if middle or high, N0 (or cN1 if high), mrf−, no EMVI: surgery (TME) alone†† If poor prognostic signs (CRM+, N2): postoperative chemotherapy or chemoradiotherapy	Extent of lymph node dissection is determined on the preoperative clinical findings and on the extent of lymph node metastases and depth of tumour invasion pTis: D0 or D1 dissection if insufficient accuracy of preoperative diagnosis of invasion depth pT1: D2 dissection cT2: D2 or D3 dissection cT3, cT4a, cT4b: D3 dissection cN+: D3 dissection
Unresectable‡	T3–4 N0 or Tany N1–2 or locally unresectable or medically inoperable: neoadjuvant therapy (CRT, RT or chemotherapy) followed by primary surgery** and adjuvant treatment¶ Tany Nany M1 unresectable metastases or medically inoperable (symptomatic): primary treatment (combination systemic chemotherapy or infusional 5‐FU/RT or bolus 5‐FU/RT or capecitabine/RT or resection of the involved rectal segment or diverting ostomy or stenting or short‐course RT (not recommended for T4)) followed by systemic therapy# Tany Nany M1 unresectable metastases or medically inoperable (asymptomatic): systemic therapy#	cT2 very low, cT3 mrf − (unless cT3a (b) and mid or high rectum), N1–2, EMVI+, limited cT4a N0: preoperative RT or CRT followed by TME (wait‐and‐see in high‐risk patients for surgery if CRT and clinical complete remission‡‡) cT3mrf+, cT4a,b, lateral node+: preoperative CRT followed by surgery (TME + more extended surgery if needed) (RT with surgery delay in elderly or in patients with severe co‐morbidity)	TME or tumour‐specific mesorectal excision (TSME) Lateral lymph node dissection is indicated when the lower border of the tumour is located distal to the peritoneal reflection and the tumour has invaded beyond the muscularis propria
Additional comments	**Surgery should be 5–12 weeks after full‐dose 5·5‐week neoadjuvant chemoradiotherapy† TME should extend 4–5 cm below distal edge of tumours; in distal rectal cancers (< 5 cm from anal verge) negative distal bowel margin of 1–2 cm may be acceptable† Extended lymph node resection is not indicated in the absence of clinically suspected nodes†	††For tumours situated in the upper third, partial mesorectal excision can be carried out with a mesorectal margin of ≥ 5 cm distal to the tumour TME implies that all mesorectal fat, including all lymph nodes, should be excised A good TME without damaging the rectal fascia surrounding the mesorectal fat and rectum is prognostically relevant If an abdominoperineal excision is planned, the dissection from above must be stopped at the tip of the coccyx and be continued from below ‡‡If no tumour can be detected and/or no viable tumour cells are found (i.e. a clinical or a pathological complete response is achieved), no further therapy is provided (organ preservation) and the patient is monitored closely for at least 5 years	Urinary function and male sexual function may be impaired after lateral dissection, even when the autonomic nervous system is preserved completely

Adapted with permission from the National Comprehensive Cancer Network (NCCN) Clinical Practice Guidelines in Oncology (NCCN Guidelines^®^) for Guideline Rectal Cancer 03.13.2017. © 2017 National Comprehensive Cancer Network, Inc. All rights reserved. The NCCN Guidelines^®^ and illustrations herein may not be reproduced in any form for any purpose without the express written permission of NCCN. To view the most recent and complete version of the NCCN Guidelines^®^, go online to http://nccn.org. The NCCN Guidelines^®^ are a work in progress that may be refined as often as new significant data becomes available. NCCN makes no warranties of any kind whatsoever regarding their content, use or application, and disclaims any responsibility for their application or use in any way.

* NCCN Recommendation 1;

† NCCN Recommendation B1;

‡ NCCN Recommendation 2;

§ NCCN Recommendation 6;

¶ NCCN Recommendation 5;

# NCCN Recommendation 7. ESMO, European Society for Medical Oncology; JSCCR, Japanese Society for Cancer of the Colon and Rectum; ERUS, endoscopic rectal ultrasonography; VI, vascular invasion; SM/sm, submucosa; CBC, complete blood count; CEA, carcinoembryonic antigen; US, ultrasonography; CRM, circumferential resection margin; MDT, multidisciplinary team; 5‐FU, 5‐fluorouracil; RT, radiotherapy; mrf, mesorectal fascia; EMVI, extramural vascular invasion; TME, total mesorectal excision; CRT, chemoradiotherapy.

### Rectal cancer (appropriate for resection)

Both NCCN and ESMO state a formal pretreatment work‐up, whereas JSCCR does not. NCCN recommends a complete blood count, chemistry profile, carcinoembryonic antigen (CEA) measurement and colonoscopy, whereas ESMO advises sigmoidoscopy (either rigid or flexible), endoscopic ultrasonography and circumferential resection margin evaluation (*Table* 1). Both NCCN and ESMO advise the use of MRI in patient work‐up.

NCCN considers transanal excision only for T1 N0 non‐fixed tumours (stage I) that are less than 3 cm in size, occupy less than 30 per cent of the circumference of the bowel and lie within 8 cm of the anal verge; advises transabdominal surgery without neoadjuvant therapy for T1–2 N0 tumours (stage I) that do not meet the previous criteria; and recommends neoadjuvant treatment, followed by surgery after 5–12 weeks, then adjuvant therapy, for all resectable disease from stage II to IV. In contrast, ESMO suggests surgery alone even for cT3a N1 high tumours, leaving postoperative treatment for those with poor prognostic features.

In consideration of local invasion (T3 mesorectal fascia (mrf) +/−, T4a–b, extramural vascular invasion (EMVI) +), both NCCN and ESMO agree on neoadjuvant treatment followed by surgery and then postoperative therapy. ESMO also suggests a ‘deferred surgery’ policy for patients at high surgical risk.

NCCN advises palliative surgery in the setting of symptomatic patients with unresectable metastatic disease, whereas neither ESMO nor JSCCR describes this event.

The same criteria for TME are shared between NCCN and ESMO (mesorectal margin of 5 cm from the tumour distal edge), whereas JSCCR focuses more on the extent of lymph node dissection, which is based on the perceived spread of lymph node metastases and depth of tumour invasion (from D0–1 for pTis to D3 for cT3–4a/b or cN+). JSCCR also recommends lateral lymph node dissection for tumours where the lower border lies distal to the peritoneal reflection or there is invasion beyond the muscularis propria.

Conversely, NCCN does not recommend extended lymph node dissection unless suspicious nodes are present, whereas ESMO highlights the importance of radical resection of mesorectal fat, including all lymph nodes (*Table* 1).

### Postoperative (adjuvant) treatment after surgery

There is substantial agreement between guidelines on adjuvant chemotherapy administration for stage III disease.

Before chemotherapy, all three guidelines agree on gene testing of *RAS* status. Both NCCN and ESMO also recommend assessment for *BRAF* V600E mutation. ESMO alone advises microsatellite instability testing in the setting of metastatic disease, and JSCCR is the only one to advise UDP‐glucuronosyltransferase1A1 (UGT1A1) phenotyping. Mismatch repair and dihydropyrimidine dehydrogenase deficiency testing are of secondary importance for NCCN and ESMO respectively.

Only NCCN recommends chemoradiotherapy (as an alternative to transabdominal resection) for T1 NX tumours with high‐risk features and for T2 NX found after transanal excision.

For stage II disease, both NCCN and ESMO recommend postoperative chemotherapy and/or radiotherapy. Although specific regimens have not been stated, ESMO follows precise principles of administration in these patients and has recently questioned its routine use for pT3 N0 tumours. According to JSCCR, the usefulness of adjuvant chemotherapy has not been proven for stage II rectal cancer. Furthermore, NCCN recommends systemic treatment when there are contraindications to surgery, along with possible observation.

For stage III disease, all three guidelines recommend adjuvant treatment. NCCN and JSCCR agree on most of the standard regimens and on the length of the administration period (6 months preferred). NCCN suggests that there is no benefit for patients older than 70 years from oxaliplatin, whereas JSCCR advises adjuvant chemotherapy to patients aged 70 years and above with good performance status, adequate organ function and no postoperative complications (*Table* 2).

**Table 2:** Postoperative (adjuvant) treatment after surgery

Topic	NCCN	ESMO	JSCCR
Recommended gene testing for metastatic disease*	*KRAS* mutation *NRAS* mutation *BRAF* mutation—V600E— MSI testing (if personal history of colorectal cancer) MMR testing (if personal history of colorectal cancer)	*RAS* mutational status (at time of diagnosis) *BRAF* mutational status—V600E—(alongside assessment of *RAS*) MSI testing (in metastatic disease setting) DPD deficiency testing (option) UGT1A1 phenoyping (option)	*RAS* type MSI testing (only for patients with suspected Lynch syndrome) UGT1A1 genetic polymorphism
T1–2, N0, M0 stage I	*After transanal excision* †: T1 NX without high‐risk features§: observe T1 NX with high‐risk features or T2 NX: transabdominal resection or chemo/RT (capecitabine/RT or infusional 5‐FU/RT (preferred) or bolus 5‐FU/leucovorin/RT) followed by observation, transabdominal resection (chemo can also be considered after resection) or consider chemotherapy (FOLFOX or CAPEOX (preferred) or 5‐FU/leucovorin or capecitabine) *After transabdominal resection* ‡: pT1–2 N0 M0: observe	None	None
T3–4, N0, M0 stage II‡	Observation Chemotherapy: FOLFOX or CAPEOX (preferred) or 5‐FU/leucovorin or capecitabine; or FOLFOX or CAPEOX (preferred) or 5‐FU/leucovorin or capecitabine; then capecitabine/RT or infusional 5‐FU/RT (preferred) or bolus 5‐FU/leucovorin/RT; then FOLFOX or CAPEOX (preferred) or 5‐FU/leucovorin or capecitabine; or Infusional 5‐FU/RT (preferred) or capecitabine or bolus 5‐FU/leucovorin/RT, followed by FOLFOX or CAPEOX (preferred) or 5‐FU/leucovorin or capecitabine No benefit to adding OX in patients aged > 70 years	Postoperative CRT with about 50 Gy, 1·8–2·0 Gy/fraction with concomitant fluoropyrimidine‐based chemotherapy Routine use has been questioned for all pT3 N0 tumours	Usefulness of adjuvant chemotherapy has not been proven Preoperative RT for patients with T status pathologically after surgery as diagnosed invasion depth cT3 or deeper or pN‐positive, where the existence of a surgical dissection plane positive (RM1) or penetration of the surgical dissection plane by the cancer (RMX) is unclear
T1–4, N1–2, M0 stage III‡	FOLFOX or CAPEOX (preferred) or 5‐FU/leucovorin or capecitabine; then capecitabine/RT or infusional 5‐FU/RT (preferred) or bolus 5‐FU/leucovorin/RT; then FOLFOX or CAPEOX (preferred) or 5‐FU/leucovorin or capecitabine; or Infusional 5‐FU/RT (preferred) or capecitabine or bolus 5‐FU/leucovorin/RT, followed by FOLFOX or CAPEOX (preferred) or 5‐FU/leucovorin or capecitabine No benefit to adding OX in patients aged > 70 years	Postoperative CRT with about 50 Gy, 1·8–2·0 Gy/fraction with concomitant fluoropyrimidine‐based chemotherapy	Recommended therapies: 5‐FU + l‐LV UFT + LV Capecitabine FOLFOX CAPEOX S‐1 In patients aged 70 years or more postoperative adjuvant chemotherapy is recommended if their PS is good, the function of main organs is adequate and there are no complications
Additional comments	§Positive margins, lymphovascular invasion, poorly differentiated tumours or sm3 invasion† 6‐months' perioperative treatment preferred‡	Indications: Positive CRM Perforation in the tumour area Defects in the mesorectum High risk of recurrence If preoperative radiotherapy has not been given	Indications for adjuvant chemotherapy: Stage III cancer for which R0 resection has been performed Peripheral blood neutrophil count > 1500/mm^3^; platelet count > 100 000/mm^3^ Total bilirubin < 2·0 mg/dl; AST/ALT < 100 units/l Serum creatinine concentration no higher than upper limit of normal range PS 0 or 1 Patient has recovered from postoperative complications In principle, administration period is 6 months

Adapted with permission from the National Comprehensive Cancer Network (NCCN) Clinical Practice Guidelines in Oncology (NCCN Guidelines^®^) for Guideline Rectal Cancer 03.13.2017. © 2017 National Comprehensive Cancer Network, Inc. All rights reserved. The NCCN Guidelines^®^ and illustrations herein may not be reproduced in any form for any purpose without the express written permission of NCCN. To view the most recent and complete version of the NCCN Guidelines^®^, go online to http://nccn.org. The NCCN Guidelines^®^ are a work in progress that may be refined as often as new significant data becomes available. NCCN makes no warranties of any kind whatsoever regarding their content, use or application, and disclaims any responsibility for their application or use in any way.

* NCCN Recommendation A5;

† NCCN Recommendation 3;

‡ NCCN Recommendation 4. ESMO, European Society for Medical Oncology; JSCCR, Japanese Society for Cancer of the Colon and Rectum; MSI, microsatellite instability; MMR, mismatch repair; DPD, dihydropyrimidine dehydrogenase; UGT1A1, UDP‐glucuronosyltransferase 1A1; RT, radiotherapy; 5‐FU, 5‐fluorouracil; FOLFOX, 5‐fluorouracil–leucovorin–oxaliplatin; CAPEOX, capecitabine–oxaliplatin; OX, oxaliplatin; CRT, chemoradiotherapy; l‐LV, levoleucovorin; UFT, tegafur–uracil; S‐1, tegafur–gimeracil–oteracil; PS, performance status; sm, submusoca; CRM, circumferential resection margin; AST, aspartate aminotransferase; ALT, alanine aminotransferase.

### Postoperative surveillance (follow‐up)

Both NCCN and JSCCR recommend a precise, systematic follow‐up, whereas ESMO advises a more flexible, patient‐tailored one.

NCCN divides follow‐up between early and more advanced stages (II–III and IV). Complete colonoscopy at 1 year is suggested for all stages, to be repeated at 3 years and then every 5 years, unless advanced adenoma (villous polyp, size greater than 1 cm and/or high‐grade dysplasia) is found. For patients treated by transanal excision, proctoscopy with endoscopic ultrasonography or MRI with contrast is advised every 3–6 months for the first 2 years, then every 6 months for up to 5 years.

Both ESMO and JSCCR divide recommendations between stage I–III and stage IV disease. Although the plan for stage I–III disease is not well defined, it broadly consists of clinical assessment every 6 months for 2 years and history and colonoscopy every 5 years. ESMO advises intensive follow‐up for patients with stage IV disease, with evaluation (history, physical examination, CEA, CT and/or MRI) every 2–3 months, especially if chemotherapy is undertaken.

The JSCCR‐recommended follow‐up schedule is more frequent than that proposed by NCCN, at least for the first years of surveillance, with annual colonoscopy and examination with tumour markers every 3 months for the first 3 years, along with CT every 6 months for the first 3 years (or 5 years in stage III disease). JSCCR alone suggests digital rectal examination every 6 months for 3 years, and establishes the duration of the postoperative surveillance as 5 years, on the basis that 95 per cent of recurrences are detected within 5 years of surgery.

None of the three guidelines gives indications for PET (*Table* 3).

**Table 3:** Postoperative surveillance (follow‐up)

Pathological stage	NCCN	ESMO	JSCCR
Stage I with full surgical staging*	Colonoscopy at 1 year If advanced adenoma†, repeat in 1 year If no advanced adenoma†, repeat in 3 years, then every 5 years	Minimum provisional recommendation: Clinical assessment every 6 months for 2 years Colonoscopy within the first year if not done at the time of diagnostic work‐up History and colonoscopy with resection of colonic polyps every 5 years up to age 75 years Clinical, laboratory and radiological examinations are restricted to patients with suspicious symptoms	Interview and examination every 3 months for 3 years, then every 6 months for a total of 5 years Tumour markers every 3 months for 3 years, then every 6 months for a total of 5 years Digital rectal examination every 6 months for 3 years Chest/abdominal/pelvic CT every 6 months for 3 years, then annually for a total of 5 years (for stage III every 6 months for 5 years) Colonoscopy annually for 3 years
Stage II* Stage III*	History and physical every 3–6 months for 2 years, then every 6 months for a total of 5 years CEA every 3–6 months for 2 years, then every 6 months for a total of 5 years Chest/abdominal/pelvic CT every 6–12 months (category 2B for frequency < 12 months) for a total of 5 years Colonoscopy in 1 year (except if no preoperative colonoscopy due to obstructing lesion, colonoscopy in 3–6 months): if advanced adenoma, repeat in 1 year; if no advanced adenoma, repeat in 3 years, then every 5 years Proctoscopy (with EUS or MRI with contrast every 3–6 months for 2 years, then every 6 months for a total of 5 years (for patients treated with transanal excision only)) PET–CT scan is not recommended		
Stage IV*	The same as for stage II–III plus chest/abdominal/pelvic CT every 3–6 months (category 2B for frequency < 12 months) for 2 years, then every 6–12 months for a total of 5 years	History, physical examination, CEA and CT (or MRI) are recommended after 2–3 months during palliative chemotherapy Patients to be re‐evaluated every 2–3 months if chemotherapy is continued No evidence for the evaluation using PET In patients who had complete resection of metastatic disease, more intensive follow‐up should be considered CEA and CT at intervals of 3–6 months during the first 3 years can be recommended	The same as for stage III For R1 resection, close surveillance schedule should be planned
Additional comments	†Villous polyp, polyp > 1 cm or high‐grade dysplasia*		The duration of surveillance is 5 years after surgery (more than 80% of recurrences are detected within 3 years of surgery, and more than 95% within 5 years) More than 95% of anastomotic recurrences are detected within 3 years of surgery

Adapted with permission from the National Comprehensive Cancer Network (NCCN) Clinical Practice Guidelines in Oncology (NCCN Guidelines^®^) for Guideline Rectal Cancer 03.13.2017. © 2017 National Comprehensive Cancer Network, Inc. All rights reserved. The NCCN Guidelines^®^ and illustrations herein may not be reproduced in any form for any purpose without the express written permission of NCCN. To view the most recent and complete version of the NCCN Guidelines^®^, go online to http://nccn.org. The NCCN Guidelines^®^ are a work in progress that may be refined as often as new significant data becomes available. NCCN makes no warranties of any kind whatsoever regarding their content, use or application, and disclaims any responsibility for their application or use in any way.

* NCCN Recommendation 8.

ESMO, European Society for Medical Oncology; JSCCR, Japanese Society for Cancer of the Colon and Rectum; CEA, carcinoembryonic antigen; EUS, endoscopic ultrasonography.

### Synchronous metastases/locally invasive disease

All three guidelines recommend curative resection of the primary tumour and distant metastases whenever possible (*Table* 4).

**Table 4:** Metastatic cancer

Disease	NCCN	ESMO	JSCCR
Synchronous metastases/locally invasive	Resectable metastases†† Pathway 1: Combination chemotherapy (2–3 months): FOLFIRI or FOLFOX or CAPEOX; followed by Staged or synchronous resection and/or local therapy for metastases and resection of rectal lesion; followed by Infusional 5‐FU/pelvic RT or capecitabine/RT (preferred) or bolus 5‐FU + leucovorin/pelvic RT; or Infusional 5‐FU/pelvic RT or capecitabine/RT (preferred) or bolus 5‐FU + leucovorin/pelvic RT or short‐course RT (not recommended for T4 tumours); followed by Staged or synchronous resection and/or local therapy for metastases and resection of rectal lesion; followed by Systemic therapy Pathway 2: Infusional 5‐FU/pelvic RT or capecitabine/RT (preferred) or bolus 5‐FU + leucovorin/pelvic RT or short‐course RT (not recommended for T4 tumours); followed by Staged or synchronous resection and/or local therapy for metastases and resection of rectal lesion; followed by FOLFOX or CAPEOX (preferred) or 5‐FU/leucovorin or capecitabine Unresectable metastases† Symptomatic: Combination systemic therapy Infusional 5‐FU/RT or bolus 5‐FU/RT or capecitabine/RT Resection of involved rectal segment Diverting ostomy Stenting Short‐course RT (not recommended for T4 tumours); followed by Systemic therapy Asymptomatic: Systemic therapy T3 N0 or Tany N1–2 or T4 and/or locally unresectable or medically inoperable‡	Historical groups for treatment stratification Group 0: primary technically R0‐resectable liver or lung metastases and non‐biological relative contraindications Surgery; or 5 × 5 Gy followed by combination chemotherapy; evaluation after 6–8 weeks and surgery after about 3 months (if unclear prognostic situation)*; followed by Postoperative chemotherapy for up to 6 months *Consider perioperative chemotherapy 3 months before and 3 months after surgery with FOLFOX or CAPEOX Group 1: potentially resectable metastatic disease with curative intention Downsizing by induction chemotherapy with cytotoxic doublet (FOLFOX or FOLFIRI) or triplet (FOLFOXIRI) +/− bevacizumab or anti‐EGFR antibodies (in patients with *RAS* wt); re‐evaluation/assessment of response every 2 months; followed by Secondary surgery (3–4 weeks from the last cycle of chemotherapy +/− cetuximab, or 6 weeks following chemotherapy plus bevacizumab) Consider also short‐course radiotherapy with combination chemotherapy starting 11–18 days later if primary tumour locally advanced (surgery 5–6 months after radiotherapy) The maximal response is expected after 12–16 weeks of therapy Oligometastatic disease (OMD): existence of metastases at up to 2 or occasionally 3 sites and 5 or sometimes more lesions, predominantly visceral and occasionally lymphonodal Treatment strategies based on the possibility of achieving complete ablation using surgical resection and/or local ablative treatment (LAT)	Treatment strategies: If both distant metastases and primary tumour are resectable, curative resection of primary tumour is performed, and resection of distant metastases considered If distant metastases are resectable, but the primary tumour is unresectable, resection is not performed and another treatment method is selected If distant metastases are unresectable, but the primary tumour is resectable, the indication for the resection of the primary is determined, based on clinical symptoms and the impact on prognosis Indication criteria for hepatectomy: Patient capable of tolerating surgery The primary tumour has been controlled or can be controlled The metastatic liver tumour can be completely resected No extrahepatic metastases or they can be controlled The function of the remaining liver will be adequate Systemic therapy considered for patients with unresectable liver metastases whose general condition can be maintained at a certain level or higher (PS0 to PS2) If PS ≥ 3 or no effective chemotherapy, then best supportive care Indication criteria for pneumonectomy: Patient capable of tolerating surgery Primary tumour has been controlled or can be controlled Metastatic lung tumour can be resected completely No extrapulmonary metastases or they can be controlled Function of the remaining lung will be adequate Systemic therapy considered for patients with unresectable lung metastases whose general condition can be maintained at a certain level or higher Consider stereotactic body RT if the primary tumour and extrapulmonary metastases are controlled or can be controlled and ≤ 3 lung metastases within 5 cm in diameter
	Pathway 1: Chemo/RT (capecitabine/long‐course RT or infusional 5‐FU/long‐course RT (preferred) or bolus 5‐FU/leucovorin/long‐course RT); or RT (short‐course RT); followed by Transabdominal resection; followed by FOLFOX or CAPEOX or 5‐FU/leucovorin or capecitabine If resection contraindicated: systemic therapy Pathway 2: Chemotherapy (FOLFOX or CAPEOX (preferred) or 5‐FU/leucovorin or capecitabine); followed by Capecitabine/RT or infusional 5‐FU/RT (preferred) or bolus 5‐FU/leucovorin/RT; followed by Transabdominal resection If resection contraindicated: systemic therapy	Systemic therapy is the standard of care The best local treatment to be selected from a toolbox of procedures, according to disease localization, treatment goal, treatment‐related morbidity and patient‐related factors Group 2: disseminated disease, technically never/unlikely resectable intermediate intensive treatment Very active first‐line treatment with a high likelihood of inducing metastases regression: FOLFOX or FOLFIRI (also FOLFOXIRI) in combination with bevacizumab or anti‐EGFR antibody (in patients with *RAS* wt) Re‐evaluation/assessment of response every 2–3 months and continue until sufficient regression; possibly followed by 5 × 5 Gy RT, surgery and adjuvant chemotherapy Group 3: never‐resectable metastatic disease Prevention of tumour progression and prolongation of life with minimal treatment burden: cytotoxic +/− biological targeted agent or escalation strategy starting with an FP +/− bevacizumab On progression: oxaliplatin‐ or irinotecan‐based combination with a biological targeted agent Revised groups for treatment stratification Group 1 fit patients Clinical presentation: Conversion and achievement of NED Impending clinical threat, impending organ dysfunction and severe (disease‐related) symptoms Treatment goal: Cytoreduction, followed by R0 resection, NED achieved by LAT Improvement of symptoms and avoidance of rapid evolution and prolonged survival Group 2 fit patients Clinical presentation: Asymptomatic No impending clinical threat Resection not an option Treatment goal: Disease control and prolonged survival Unfit patients Best supportive care Palliative	
		Continuum of care: 4–6 months of first‐line induction therapy 4–6 (–8) months of maintenance therapy or no treatment after resection and/or ablation 3 months reintroduction 5–7 months of second‐line therapy Treatment break 3 months of third‐line therapy Potentially fourth‐line therapy A few months of rechallenge of initial induction or first‐line therapy A few months of best supportive care only Aim: 70–80% of fit patients to receive second‐line therapy and 50–60% of fit patients to receive third‐line therapy	
Metachronous metastases/local recurrence	Resectable metachronous metastases§ No previous chemotherapy Resection (preferred) and/or local therapy; followed by FOLFOX or CAPEOX (preferred) or FLOX or capecitabine or 5‐FU/leucovorin; or Neoadjuvant chemotherapy (2–3 months) – FOLFOX or CAPEOX (preferred) or FLOX or capecitabine or 5‐FU/leucovorin; followed by Resection (preferred) and/or local therapy If no growth on neoadjuvant chemotherapy: reinitiate neoadjuvant chemotherapy or FOLFOX or observation If growth on neoadjuvant chemotherapy: systemic therapy +/− biological therapy (category 2B) or observation Previous chemotherapy Resection (preferred) and/or local therapy; followed by Observation or systemic therapy +/− biological therapy (category 2B); or Neoadjuvant chemotherapy (2–3 months) – FOLFOX or CAPEOX (preferred) or FLOX or capecitabine or 5‐FU/leucovorin; followed by Resection (preferred) and/or local therapy If no growth on neoadjuvant chemotherapy: reinitiate neoadjuvant chemotherapy or FOLFOX or observation If growth on neoadjuvant chemotherapy: systemic therapy +/− biological therapy (category 2B) or observation	Local recurrences If RT has not been given in the primary situation, patients with recurrence should receive preoperative radiotherapy with concomitant chemotherapy In patients previously irradiated, additional RT, external and/or IORT or different brachytherapy Attempts at radical surgery should be done 6–10 weeks after RT In patients with previous RT for whom salvage surgery is not an option, systemic palliative chemotherapy may be tried	Local recurrence Resection should be considered for local recurrence of rectal cancer (anastomotic and intrapelvic recurrence) when R0 resection is considered possible RT and systemic chemotherapy are considered for unresectable recurrences Metachronous metastases If recurrence is observed in a single organ and complete resection of the recurrent tumour(s) is possible, resection is strongly considered If recurrence is observed in more than a single organ, resection can be considered if the tumours in all of the organs are resectable
	Unresectable metachronous metastases¶ Previous adjuvant FOLFOX/CAPEOX within past 12 months FOLFIRI +/−(bevacizumab orziv‐aflibercept or ramucirumab) oririnotecan +/− (bevacizumab orziv‐aflibercept or ramucirumab) orFOLFIRI + (cetuximab or panitumumab(*KRAS*/*NRAS* wt only)) or irinotecan +/−(cetuximab or panitumumab (*KRAS*/*NRAS* wt only)) or (nivolumab orpembrozilumab (dMMR/MSI‐H only)) Followed by re‐evaluation for resectabilityevery 2 months If converted to resectable: resection; followed by systemic therapy +/− biological therapy (category 2B) or observation If remains unresectable: systemic therapy		
	Previous adjuvant FOLFOX/CAPEOX >12 months; previous 5‐FU/leucovorin or capecitabine; no previous chemotherapy Systemic therapy, followed by re‐evaluation for resectability every 2 months If converted to resectable: resection, followed by systemic therapy +/− biological therapy (category 2B) or observation If remains unresectable: systemic therapy Isolated pelvic/anastomotic recurrence# Potentially resectable Resection, followed by capecitabine + RT or infusional 5‐FU + RT or bolus 5‐FU + RT; or Preoperative capecitabine + RT or infusional 5‐FU + RT or bolus 5‐FU + RT; followed by Resection +/− IORT Unresectable Chemotherapy +/− RT		
Peritoneal disease	Complete cytoreductive surgery and/or intraperitoneal chemotherapy can be considered in experienced centres for selected patients with limited peritoneal metastases for whom R0 resection can be achieved**	In selected patients, complete cytoreductive surgery and HIPEC may provide prolonged survival when carried out in experienced high‐volume centres The efficacy depends on the extent of peritoneal dissemination and is scored using the PCI, which is the main prognostic factor Involvement of the lower ileum is a negative prognostic factor Cytoreductive surgery is particularly effective in patients with low‐volume peritoneal disease (PCI < 12) and no evidence of systemic disease	If the resection is not significantly invasive, the peritoneal dissemination should be resected at the same time as the primary tumour
Additional comments	Re‐evaluation for resection in otherwise unresectable patients after after 2 months of preoperative chemotherapy and every 2 months thereafter†† When considering whether disease has been converted to resectable, all original sites need to be amenable to resection††	Biological targeted agents in second‐line therapy: in patients who started with bevacizumab as first line, the options are bevacizumab, aflibercept and, in *RAS* wt patients, cetuximab or panitumumab Contraindications to hepatic resection: Technical Absolute: impossibility of R0 resection with ≥ 30% liver remnant; presence of unresectable extrahepatic disease Relative: R0 resection possible only with complex procedure; R1 resection Oncological Concomitant extrahepatic disease (unresectable) Number of lesions ≥ 5 Tumour progression	Indications for systemic chemotherapy: Clinical or histopathological diagnosis confirmed Metastatic or recurrent tumour con be confirmed by imaging PS 0–2 Peripheral blood neutrophil count > 1500/mm^3^; platelet count > 100 000/mm^3^ Total bilirubin < 2.0 mg/dl; AST/ALT < 100 units/l Serum creatinine concentration no higher than upper limit of normal range Written informed consent provided Patient has no serious complications

Adapted with permission from the National Comprehensive Cancer Network (NCCN) Clinical Practice Guidelines in Oncology (NCCN Guidelines^®^) for Guideline Rectal Cancer 03.13.2017. © 2017 National Comprehensive Cancer Network, Inc. All rights reserved. The NCCN Guidelines^®^ and illustrations herein may not be reproduced in any form for any purpose without the express written permission of NCCN. To view the most recent and complete version of the NCCN Guidelines^®^, go online to http://nccn.org. The NCCN Guidelines^®^ are a work in progress that may be refined as often as new significant data becomes available. NCCN makes no warranties of any kind whatsoever regarding their content, use or application, and disclaims any responsibility for their application or use in any way.

†† NCCN Recommendation 6;

† NCCN Recommendation 7;

‡ NCCN Recommendation 5;

§ NCCN Recommendation 10;

¶ NCCN Recommendation 11;

# NCCN Recommendation 9;

** Manuscript 32;

†† NCCN Recommendation B2. ESMO, European Society for Medical Oncology; JSCCR, Japanese Society for Cancer of the Colon and Rectum; FOLFIRI, 5‐fluorouracil–leucovorin–irinotecan; FOLFOX, 5‐fluorouracil–leucovorin–oxaliplatin; CAPEOX, capecitabine–oxaliplatin; 5‐FU, 5‐fluorouracil; RT, radiotherapy; EGFR, epithelial growth factor receptor; wt, wild‐type; FOLFOXIRI, 5‐fluorouracil–leucovorin–oxaliplatin–irinotecan; FP, fluoropyrimidine; NED, no evidence of disease; LAT, local ablative treatment; PS, performance status; FLOX, 5‐fluorouracil–oxaliplatin; MMR, mismatch repair; MSI, microsatellite instability; IORT, intraoperative radiotherapy; HIPEC, hyperthermic intraperitoneal chemotherapy; PCI, peritoneal cancer index; AST, aspartate aminotransferase; ALT, alanine aminotransferase.

NCCN distributes patients into three categories: those with resectable metastases, unresectable metastases or local invasion. For patients with resectable metastases, there are two pathways, both characterized by neoadjuvant treatment, followed by surgery and then adjuvant therapy. Surgery is intended as staged or synchronous resection and/or local therapy for metastases and resection of the primary. The choice for preoperative and postoperative therapy is 5‐fluorouracil or capecitabine in conjunction with radiotherapy or combination chemotherapy (FOLFOX (5‐fluorouracil–leucovorin–oxaliplatin), CAPEOX (capecitabine–oxaliplatin)) or systemic therapy (combination chemotherapy plus biological targeted agents). For patients with unresectable metastases, the presence of symptoms is an indication for palliative surgery or stenting, whereas systemic therapy represents the backbone of treatment. In consideration of local invasion, there are again two pathways, distinguished by the timing of combination chemotherapy (*Table* 4).

ESMO divides patients into two groups (historical and revised) for treatment stratification. In the former, for group 0 patients (R0 resection technically achievable), radical surgery and perioperative chemotherapy are suggested. Surgery is advised in the same way for group 1 patients (potentially resectable disease with curative intention or oligometastatic disease), after neoadjuvant chemoradiotherapy followed by adjuvant chemotherapy. Conversely, for group 2 patients (disseminated disease, unlikely to be resectable) active first‐line  treatment is recommended to induce regression, followed by  assessment every 2–3 months. Chemotherapy with the intention of  prolonging life and preventing tumour progression is indicated for group 3 patients (never resectable disease). Considering the revised groups, there are fit patients in group 1 for whom radical treatment is the goal, fit patients in group 2 eligible for disease control and prolonged survival, and unfit patients who might be referred for palliative and/or best supportive care. ESMO is the only guideline to state a scheduled continuum of care and to list technical and oncological contraindications to hepatic resection.

JSCCR discourages resection of distant metastases if the primary tumour is unresectable, whereas it suggests resection of the primary in patients with unresectable metastases, based on clinical symptoms and prognostic impact. Furthermore, it establishes criteria for both hepatectomy and pneumonectomy, as well as stereotactic body radiotherapy. JSCCR also provides general indications for systemic chemotherapy (*Table* 4).

### Metachronous metastases/local recurrence

All three guidelines agree that surgical resection of metachronous metastases or local recurrence should be performed whenever possible.

NCCN guidelines describe three different scenarios: resectable metachronous metastases, unresectable metachronous metastases and pelvic/anastomotic recurrence (local recurrence) (*Table* 4).

There are two possible pathways for resectable metachronous metastases based on previous administration of chemotherapy. If chemotherapy has already been given, resection or local therapy followed by adjuvant combination treatment is suggested. Alternatively, neoadjuvant combination therapy followed by resection or local treatment can be offered. In case of metastatic growth during neoadjuvant treatment, a switch to systemic or biological therapy is advised. Where chemotherapy has been used previously, recommendations are similar, although systemic/biological therapy and observation are given more prominence.

The treatment strategy for unresectable metachronous metastases depends on previous adjuvant therapy. If FOLFOX or CAPEOX has been given during the past year, treatment with FOLFIRI with or without a biological agent is advised, with the aim of conversion to resectable disease (re‐evaluation every 2 months) for radical surgery. If that goal is not achieved, systemic therapy is recommended. If no previous chemotherapy has been administered, systemic therapy with the aim of conversion to resectable disease is advised in the same way.

Surgery is recommended for pelvic/anastomotic recurrence if the local recurrence is amenable to resection, possibly associated with intraoperative radiotherapy (IORT), and preceded or followed by chemoradiotherapy.

ESMO guidelines do not state specific treatment for metachronous disease (*Table* 4). Despite that, radiotherapy is strongly suggested for local recurrence, and IORT and brachytherapy are also advised. Radical surgery is recommended 6–10 weeks after radiotherapy.

Similarly, JSCCR recommendations are brief: resection should be considered for local recurrence and distant metachronous metastases whenever R0 resection is considered obtainable (*Table* 4).

### Peritoneal disease

All three guidelines agree on the principle that complete cytoreductive surgery and intraperitoneal chemotherapy should be considered in experienced centres whenever R0 resection can be achieved. ESMO underlines the importance of the peritoneal cancer index (PCI) for prognosis, and regards involvement of the distal ileum as a negative prognostic factor (*Table* 4).

### Minimally invasive surgery

Laparoscopic surgical approaches are formally stated only by NCCN and JSCCR, with agreement on the importance of experience and technical skills. NCCN cites locally advanced disease, with a threatened or high‐risk circumferential margin based on staging, and emergency setting, such as acute bowel obstruction or perforation, as contraindications. JSCCR underlines the importance of tumour location, obesity and previous open abdominal surgery.

None of the guidelines mentions robotic surgery (*Table* 5).

**Table 5:** Additional considerations

	NCCN	ESMO	JSCCR
Laparoscopic surgery*	Principles: Technical expertise is required Not indicated for locally advanced disease with a threatened or high‐risk circumferential margin Not indicated for acute bowel obstruction or perforation	Not stated formally	Determined by: Surgeon's experience and skills Tumour location Degree of cancer progression Obesity History of open abdominal surgery
Robotic surgery	Not mentioned	Not mentioned	Not mentioned
Restaging†	Surgical re‐evaluation to be planned approximately 2 months after initiation of chemotherapy; for unresectable patients who continue to receive chemotherapy, surgical re‐evaluation every 2 months thereafter	In patients receiving conversion therapy it is recommended that resectability is first evaluated after 2 months of optimal treatment and again after 4 months, when the maximum tumour shrinkage is deemed to have occurred in most patients (maximal response is expected to be achieved after 12–16 weeks of therapy)	Not formally stated
Complete radiological response	Not described	MRI: reduction in size can be seen, as well as increase in fibrosis and mucous degeneration indicating response PET–CT: reduction in uptake can be seen At present, the relevance of these changes is not understood and the extent of surgery should not be modified based on this	Not described
TRG classification on MRI	Not mentioned	Not mentioned	Not mentioned
Watch and wait policy‡	This approach is not supported in the routine management of localized rectal cancer	If no tumour can be detected and/or no viable tumour cells are found after CRT (i.e. a cCR or pCR is achieved), no further therapy is provided (organ preservation) and the patient is monitored closely for at least 5 years It is then assumed that potential lymph node metastases have been eradicated in conjunction with the excellent response of the tumour This strategy has not yet been subjected to properly controlled prospective studies	Not described
Advanced primary or recurrent cancer Surgery beyond TME	Total mesorectal excision* Reduces positive radial margin rate Extend 4–5 cm below distal edge of tumours for an adequate mesorectal excision. In distal rectal cancers (< 5 cm from anal verge), negative distal bowel wall margin of 1–2 cm may be acceptable; this must be confirmed to be tumour‐free by frozen‐section examination Full rectal mobilization allows for a negative distal margin and adequate mesorectal excision Local/anastomotic recurrence (resectable)¶ Optimally managed with resection followed by adjuvant CRT or with preoperative RT and concurrent chemotherapy	Total mesorectal excision All mesorectal fat, including all lymph nodes, should be excised A good TME without damaging the rectal fascia surrounding the mesorectal fat and rectum is prognostically relevant If an abdominoperineal excision is planned, the dissection from above must be stopped at the tip of the coccyx and be continued from below The dissection plane is likely to be the most important factor for the high R1 resection rates and local recurrence rates	The principle for radical surgery is TME or tumour‐specific mesorectal excision (TSME) Lateral node dissection is often added to the TME, as lateral pelvic lymph node metastases may occur

Adapted with permission from the National Comprehensive Cancer Network (NCCN) Clinical Practice Guidelines in Oncology (NCCN Guidelines^®^) for Guideline Rectal Cancer 03.13.2017. © 2017 National Comprehensive Cancer Network, Inc. All rights reserved. The NCCN Guidelines^®^ and illustrations herein may not be reproduced in any form for any purpose without the express written permission of NCCN. To view the most recent and complete version of the NCCN Guidelines^®^, go online to http://nccn.org. The NCCN Guidelines^®^ are a work in progress that may be refined as often as new significant data becomes available. NCCN makes no warranties of any kind whatsoever regarding their content, use or application, and disclaims any responsibility for their application or use in any way.

* NCCN Recommendation B1;

† NCCN Recommendation B2 and Manuscript 34;

‡ NCCN Manuscript 23;

¶ NCCN Manuscript 49. ESMO, European Society for Medical Oncology; JSCCR, Japanese Society for Cancer of the Colon and Rectum; TRG, tumour regression grade; CRT, chemoradiotherapy; cCR, clinical complete response; pCR, pathological complete response; RT, radiotherapy; TME, total mesorectal excision.

### Restaging

NCCN and ESMO agree on re‐evaluation every 2 months in patients receiving chemotherapy with the aim of conversion to resectability. ESMO holds that maximum response is expected to be achieved after 12–16 weeks of therapy. ESMO alone envisages a complete radiological response on MRI and/or PET–CT, although the relevance of this remains unclear. JSCCR does not formally state the restaging. None of the guidelines mentions tumour regression grading (TRG) classification (*Table* 5).

### Watch‐and‐wait policy

ESMO considers a watch‐and‐wait policy for complete response after chemoradiotherapy and suggests strict monitoring for a minimum 5 years, without providing supporting evidence. NCCN does not support a watch‐and‐wait policy in the routine management of localized disease, and JSCCR does not describe this approach (*Table* 5).

### Surgery beyond total mesorectal excision

All guidelines agree that TME should be viewed as the standard for surgery, but none describes more advanced procedures such as extralevator abdominoperineal excision or total pelvic exenteration (*Table* 5).

## Discussion

There were areas of general agreement among American, European and Japanese guidelines in the management of rectal cancer. Due to the qualitative rather than quantitative nature of this study, no attempt was made to score concordance between the analysed guidelines. The aim was to highlight the main points of each guideline, in order to promote cooperations that might explain differences and to see whether these might be resolved.

Guidelines are designed to provide up‐to‐date recommendations to enable surgeons to deliver best practice. Guidelines should, consequently, analyse key points^8^. There were some key issues where there were clear differences. Regarding polyps with invasive cancer, JSCCR promoted the role of advanced endoscopic techniques (ESD and EMR) that were less considered by Western guidelines. In particular, ESD, although considered a safe and effective procedure^9^, ^10^, ^11^, has yet to achieve widespread adoption in Western countries mainly due to its long learning curve and poorly defined training processes^12^. In terms of pathology review of polyps, JSCCR alone considered tumour budding among indications for radical surgery, on the basis of evidence indicating its value as a prognostic factor^13^, ^14^, ^15^.

Regarding radical surgery, Japanese guidelines advocated lateral lymph node (or pelvic side wall) dissection whenever the lower tumour border was located distal to the peritoneal reflection^16^. Despite evidence, this remains controversial, with concerns about complications and lack of additional benefit^17^ ^18^. The wider use of chemoradiotherapy in the West probably explains the guideline differences^19^ ^20^.

Regarding postoperative chemo/radiotherapy, NCCN supported this indication for T1–2 tumours after transanal excision, mainly as an alternative to radical surgery^21^, ^22^, ^23^. In the same way, only NCCN guidelines advised adjuvant treatment for stage II cancer. Although results in terms of overall and disease‐free survival may be impressive^24^, the therapeutic decision should be taken carefully by a multidisciplinary team, balancing possible benefits and drawbacks^25^. Interestingly, neoadjuvant chemotherapy was not described by JSCCR guidelines, even though its positive outcomes have been widely demonstrated^26^, ^27^, ^28^.

For surveillance protocols, it was noteworthy that only NCCN suggested just follow‐up colonoscopy for stage I patients with full surgical staging, in accordance with other American guidelines^29^ ^30^.

With respect to the management of metastatic disease, it was interesting to note that European guidelines did not specifically assess the scenario of distant recurrent metastases, possibly reflecting the similar outcome of synchronous and metachronous disease^31^ ^32^. All three guidelines agreed that for metastatic disease, local recurrence or locally advanced disease, surgery should be attempted whenever possible.

Despite TME being regarded as the standard procedure, none of the guidelines mentioned more ‘aggressive’ surgery, as suggested by the Beyond TME Collaborative^33^, nor robotic approaches, in spite of relatively widespread adoption^34^ ^35^.

The issue of restaging in the context of patients thought initially to have unresectable metastatic disease, who have apparent responses to induction therapy, was covered only in the Western guidelines. The concepts of complete radiological response and TRG were not taken into consideration by the guidelines, despite available evidence^36^ ^37^.

Although well described, non‐operative management or deferred surgery^38^ ^39^, also referred to as a watch‐and‐wait approach, was described as a possible therapeutic option only in the ESMO guidelines; American and Japanese documents failed to mention it.

Differences between the guidelines existed, potentially reflecting the frequency with which different clinical patterns of disease present in different parts of the world. Political, economic and social contexts are also likely to be influential. Despite these considerations, specific discrepancies relating to preoperative work‐up, management of early disease, extended lymph node dissection, adjuvant treatment for early stages, and neoadjuvant therapy make these logical topics where future research could be directed profitably.
